# CGRP outflow into jugular blood and cerebrospinal fluid and permeance for CGRP of rat dura mater

**DOI:** 10.1186/s10194-021-01320-9

**Published:** 2021-09-08

**Authors:** Miriam Risch, Birgit Vogler, Mária Dux, Karl Messlinger

**Affiliations:** 1 Institute of Physiology and Pathophysiology, Friedrich-Alexander-University Erlangen-Nürnberg, Universitätsstr. 17, D-91054 Erlangen, Germany; 2 Department of Physiology, University of Szeged, Dóm tér 10, Szeged, H-6720 Hungary

**Keywords:** Calcitonin gene-related peptide, Jugular plasma, Cerebrospinal fluid, Meningeal blood flow, Primary headaches

## Abstract

**Background:**

Calcitonin gene-related peptide (CGRP) is released from activated meningeal afferent fibres in the cranial dura mater, which likely accompanies severe headache attacks. Increased CGRP levels have been observed in different extracellular fluid compartments during primary headaches such as migraine but it is not entirely clear how CGRP is drained from the meninges.

**Methods:**

We have used an in vivo preparation of the rat to examine after which time and at which concentration CGRP applied onto the exposed parietal dura mater appears in the jugular venous blood and the cerebrospinal fluid (CSF) collected from the cisterna magna. Recordings of meningeal (dural) and cortical (pial) blood flow were used to monitor the vasodilatory effect of CGRP. In a new ex vivo preparation we examined how much of a defined CGRP concentration applied to the arachnoidal side penetrates the dura. CGRP concentrations were determined with an approved enzyme immunoassay.

**Results:**

CGRP levels in the jugular plasma in vivo were slightly elevated compared to baseline values 5-20 min after dural application of CGRP (10 μM), in the CSF a significant three-fold increase was seen after 35 min. Meningeal but not cortical blood flow showed significant increases. The spontaneous CGRP release from the dura mater ex vivo was above the applied low concentration of 1 pM. CGRP at 1 nM did only partly penetrate the dura.

**Conclusions:**

We conclude that only a small fraction of CGRP applied onto the dura mater reaches the jugular blood and, in a delayed manner, also the CSF. The dura mater may constitute a barrier for CGRP and limits diffusion into the CSF of the subarachnoidal space, where the CGRP concentration is too low to cause vasodilatation.

## Background

Calcitonin gene-related peptide (CGRP) is a 37 amino acid neuropeptide that exists in two isoforms, αCGRP and βCGRP, which differ in three amino acids [1]. While αCGRP, a product of alternative splicing of the calcitonin gene, is found in the central and peripheral nervous system, where it is particularly present in sensory neurons with C and Aδ fibers, βCGRP is encoded by a second CGRP gene primarily expressed in the enteric nervous system [2]. In the trigeminovascular system, CGRP immunoreactive nerve fibers accompany dural, pial and intracerebral arteries [3–5]. These afferent fibers originate in the trigeminal ganglion and project mainly to the spinal trigeminal nucleus [6]. Activation of these nerve fibers causes CGRP release, which dilates cranial blood vessels and stimulates neuronal transmission in the spinal trigeminal nucleus [7]. The vasodilatory effect of CGRP depends on an increase in cAMP within vascular smooth muscle cells that express CGRP receptors [8].

Many studies have aimed to measure CGRP concentrations in different extracellular compartments as a measure of activation of nociceptive trigeminal afferents in preclinical models of headache and orofacial pain [9–14] as well as in clinical studies [15–18]. Elevated levels of CGRP have been found during cluster headache and migraine attacks in jugular venous blood [19–23], saliva [17] and lacrimal fluid [24, 25]. Conflicting results have been published regarding an increase in CGRP in circulating blood taken from the cubital veins [23, 26]. During massive activation of trigeminal afferents, CGRP is thought to be released from peripheral fibres of trigeminal afferents innervating meninges (dura mater and pia mater, large intracerebral arteries) and extracranial tissues, from the central terminals of these fibres in the spinal trigeminal nucleus and possibly from the neuronal somata within the trigeminal ganglion [11, 27–30]. CGRP released from trigeminal fibres outside the blood brain barrier are probably drained via intra- and extracranial venous vessels into the jugular blood [31]. It is not known if and to what extent CGRP released within the dura mater can diffuse through the meningeal neurothelium into the CSF of the subarachnoidal space [32, 33]. In contrast, CGRP from afferents innervating pial blood vessels supplying the superficial cortex and the medulla oblongata is probably directly released into the cerebrospinal fluid (CSF), because these vessels are equipped with a blood-brain barrier (BBB) [31, 34]. Likewise, CGRP released from afferents innervating cerebral blood vessels and from activated central terminals of primary afferents within the spinal trigeminal nucleus may diffuse into the CSF or is transported by lymphatic vessels into the venous system [35–37]. In addition, CGRP can be secreted together with CSF through the Pacchioni granulations into the blood of the superior sagittal sinus [38–40].

Basal CGRP concentrations in human venous blood plasma have been reported to vary widely between 20 pg/mL and more than 200 pg/mL [41, 42], which is comparable to the few existing experimental data in the rat [13, 43–46]. In migraine and cluster headache attacks, where especially meningeal afferents are assumed to be massively activated releasing their neuropeptides, the CGRP plasma concentrations in the jugular vein are roughly doubled [41, 42]. The CGRP concentrations in the CSF are approximately similar to the levels in plasma both in human and rat [10, 47] but can rise dramatically under severe intracranial disorders such as meningitis or subarachnoid bleeding [48–50]. However, the CGRP concentrations at the site of release, i.e., particularly in the meninges, are not known, and it is almost impossible to calculate them from the detected concentrations in plasma or CSF. Therefore, we set out to test in rat models ex vivo and in vivo how much of a defined concentration of CGRP applied onto the cranial dura mater diffuses through the dura and appears in the cerebrospinal fluid and the jugular plasma and how quickly this diffusion occurs. In addition, meningeal and cortical blood flow and arterial pressure were monitored as control parameters, taking into account that CGRP is a potent vasodilator.

## Methods

Animal care and all experimental procedures were performed in compliance with the guidelines for the welfare of experimental animals of the Federal Republic of Germany and the European Commission (Directive 2010/63/EU). A total of 26 Wistar rats weighing 250-470 g were used. They were bred and housed in the animal facility of the Institute of Physiology and Pathophysiology under a 12-12 h light-dark cycle with food and water ad libitum.

### In vivo experiments

**Fig. 1:**
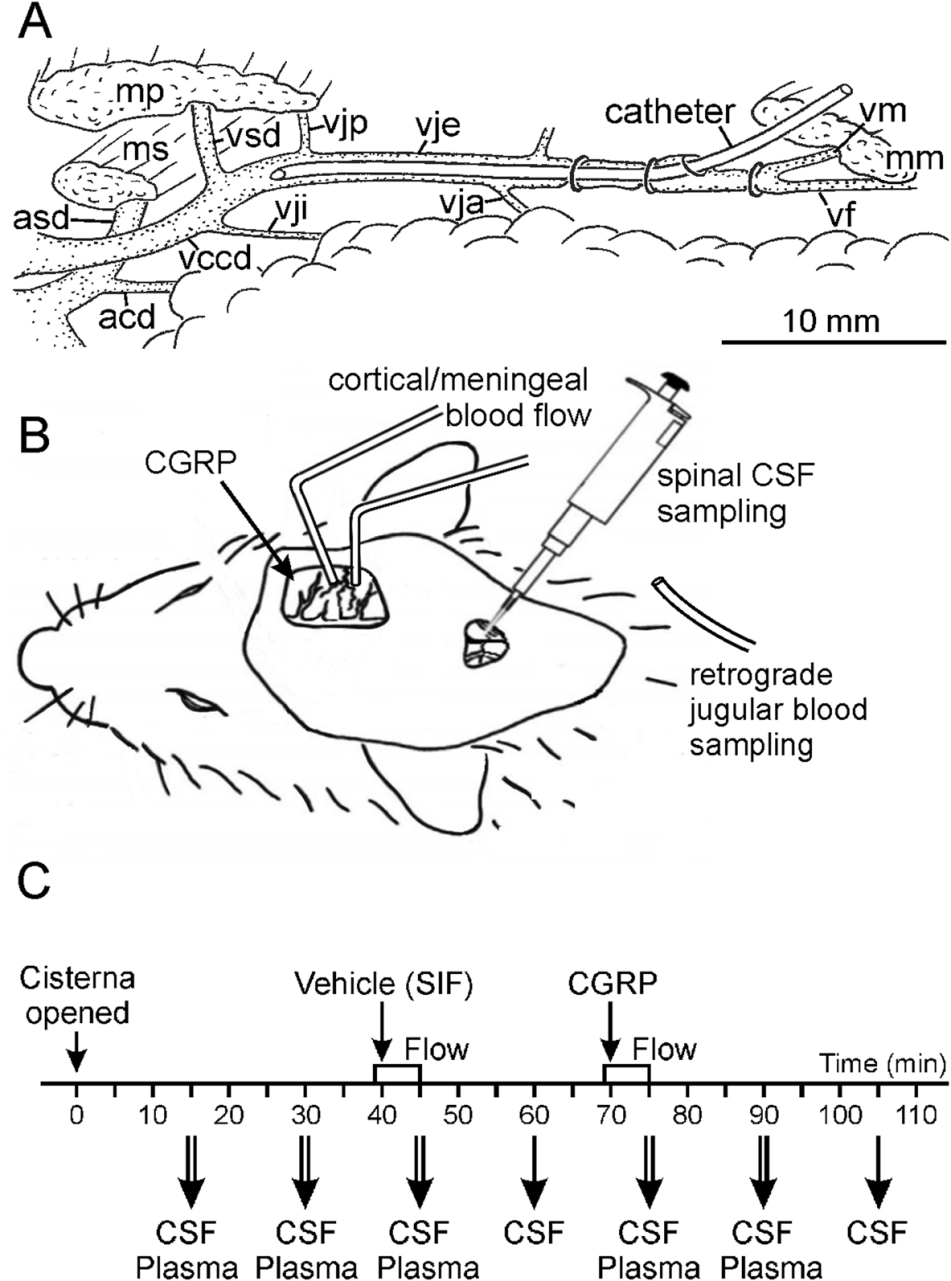
Preparation and set-up for in vivo experiments. **A.** Main blood vessels of the rat neck region of the right side with the catheter introduced into the external jugular vein for drawing blood samples. Labelling: acd, arteria carotis dextra; asd, arteria subclavia dextra; mm, mp, ms, musculus masseter, pectoralis, sternomastoideus; vccd, vena cava cranialis dextra; vf, vena facialis; vja, vje, vji, vjp, vena jugularis anterior, externa, interna, posterior; vm, vena maxillaris; vsd, vena subclavia dextra. **B.** Experimental setup. **C.** Timeline of in vivo experiments

Animals were anesthetized with 4% isoflurane (Forene, Abott, Wiesbaden, Germany) in a closed box administered with an evaporator system (Vapor 19.3, Dräger, Lübeck, Germany) and continued with 2.5% isoflurane supplied through a tight mask. After tracheotomy the animals were ventilated via a tracheal tube throughout the experiment with 2% isoflurane in oxygen-enriched room air to maintain a constant depth of anesthesia, constant blood pressure and failing motor reflexes upon noxious stimulation. A cannula connected to a pressure transducer was introduced into the exposed right femoral artery and fixed by ligatures. For systemic administration of substances, a venous catheter was inserted into the right femoral vein accordingly. In some experiments a third catheter was inserted into the exposed right external jugular vein and pushed forward close to the bifurcation of the subclavian and the internal jugular vein in order to take blood samples for measuring CGRP levels (Fig. 1A). To prevent salivation and smooth muscle spasms, atropine sulfate (Braun Melsungen AG, Melsungen, Germany, 0.5 mg/mL 1:10 with sodium chloride 0.9%) was subcutaneously injected. Throughout the experiment vital parameters such as mean blood pressure, body temperature, respiratory rate and expiratory CO_2_ levels were monitored. The body temperature was recorded with a rectal probe and kept at 37 – 37.5 °C with a feedback-controlled heating plate (TKM 0902, Föhr Medical Instruments, Frankfurt, Germany) on which the animal’s body rested. The expiratory CO_2_ was steady at 4.5-5% by modulating the ventilation frequency between 70 and 100 strokes per minute to suppress spontaneous breathing. An ointment (Bepanthen, Bayer, Leverkusen, Germany) protected the animal’s eyes. At the end of each experiment an overdose of sodium thiopental (Trapanal, Altana, Konstanz, Germany) was i.v. injected followed by exsanguination to terminate the experiment.

### Head surgery and measurement of meningeal blood flow

Animals were placed in a stereotactic frame, a snout clamp and ear bars fixed the position of the head. A median incision from the forehead to the neck exposed the skull. The parietal skull was carefully trepanned on both sides using a dental drill (KaVo Dental GmbH, Biberach, Germany) with saline cooling and a fine forceps to remove the innermost layer of the bone without breaching the dura. The exposed dura mater in the cranial windows with a size of about 6 × 4 mm was protected from drying with isotonic saline. In order to gain access to the cisterna magna, in most of the experiments the neck muscles were divided in the midline and held apart with a clamp so that the atlanto-occipital ligament with the underlying dura mater was exposed and covered with isotonic saline. After waiting 20-30 min to allow equilibration of the injured tissues, the ligament was incised and the CSF welling out was sucked off and discarded.

Blood flow was recorded by a laser Doppler system at a sampling rate of 10 Hz (DRT4, Moor Instruments, Axminster, U.K.) with needle type probes (tip diameter 0.8 mm). One probe was positioned on a branch of the middle meningeal artery and, at sufficient distance, the other on a branch of the middle cortical artery visible through the dura mater (Fig. 1B). Occasionally, the second probe was positioned over an artery of the exposed spinal medulla. The DRT4 program stored and processed perfusion data (in arbitrary units) as well as the systemic blood pressure (in mmHg). Blood flow was monitored and the experiment started only when the flow was constant, which lasted about 30 min. Changes in blood flow and blood pressure after application of substances were determined by comparing the mean flow values of each minute within the first 5 min after application with the flow during the one-minute period prior to drug application as baseline.

### Application and measurement of CGRP concentration in plasma and cerebrospinal fluid

The exposed dura mater in the cranial window was covered with 40 μl synthetic interstitial fluid (SIF) containing (in mM): 107.8 NaCl, 3.5 KCl, 0.69 MgSO_4_ · 7H_2_O, 26.2 NaHCO_3_, 1.67 NaH_2_PO_4_ · 2H_2_O, 9.64 Na-gluconate, 5.55 glucose, 7.6 sucrose and 1.53 CaCl_2_ · 2H_2_O buffered to pH 7.4 with carbogen gas (95% O_2_, 5% CO_2_). After 30 min SIF was replaced by 40 μl human ·α-CGRP at 10 μM (TOCRIS Bioscience, Wiesbaden-Nordenstadt, Germany) dissolved in SIF (Fig. 1C). Seven consecutive samples (30-60 μL) of the cerebrospinal fluid (CSF) were collected from the cisterna magna 5 min after the application of substances and then at regular intervals of 15 min as indicated. From each CSF sample one or two parts of 25 μL were taken and immediately diluted with 100 μl enzyme-immunoassay (EIA) buffer containing peptidase inhibitors (Bertin Pharma/SPIbio, Montigny le Bretonneux, France), deep-frozen and stored at − 20 °C until further analysis.

Five blood samples (200 μL each) were withdrawn through the jugular catheter immediately after CSF sampling except for the time points at 60 and 105 min (Fig. 1C). Blood samples were collected in tubes containing 0.01 mL ethylene diamine tetra-acetic acid sodium salt solution (EDTA 0.2 M; Sigma, Taufkirchen, Germany). The samples were centrifuged with 5000 rpm for 3 min, then two 25 μL fractions of the plasma supernatant were taken off with a micropipette, diluted with 100 μL EIA buffer and immediately frozen at − 20 °C.

Each of the final CSF and plasma samples was processed using the EIA for human CGRP according to the instructions of the manufacturer (Bertin Pharma/SPIbio, Montigny le Bretonneux, France). The EIA is based on a double-antibody sandwich technique with monoclonal antibodies binding the CGRP molecule; the tracer antibody is conjugated with acetylcholine esterase converting Ellman’s reagent to a colour, the absorbance of which is measured by a photo-spectrometer (Opsys MR, Dynex Technologies, Denkendorf, Germany). The assay has full cross-reactivity also for rat CGRP with a difference in the range of the intra-assay variation and is detecting both α- and β-CGRP with the same sensitivity. For plasma samples 5 pg/mL is the lower limit of detection according to the manufacturer’s information. The CGRP concentrations in the original CSF and the plasma samples were calculated in pg/mL considering the added volume of EIA buffer.

### Ex vivo experiments

**Fig. 2:**
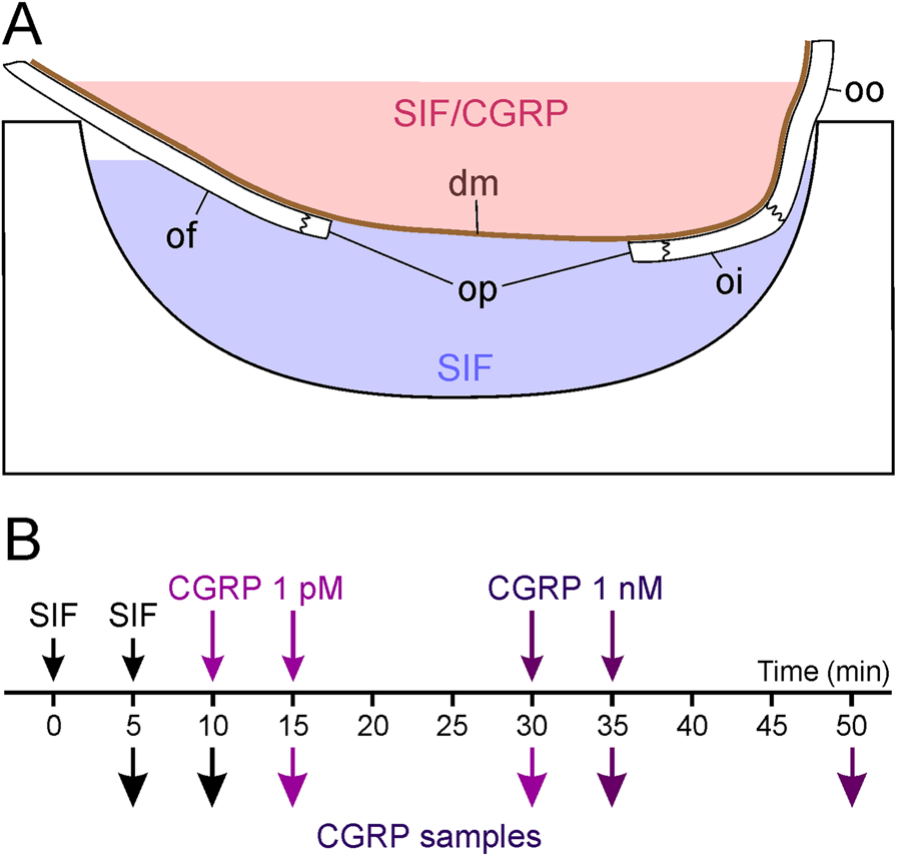
Set-up for ex vivo experiments. **A.** Apical cranium with the trepanned parietal bone shown in longitudinal section placed in the glass well. The well was filled with SIF and the cranium with SIF followed by CGRP solutions. At periods of 5 and 15 min both solutions were removed and analysed for CGRP content. SIF, synthetic interstitial fluid; dm, dura mater; of, os frontale; oi, os interparietale; oo, os occipitale; op, os parietale. B. Timeline of ex vivo experiments

Animals were exposed to 5% isoflurane in a closed box administered with an evaporator, followed by intraperitoneal injection of 180 mg/kg sodium thiopental, which induced deep anaesthesia with a minimum of vital functions (slow and flat ventilation, slow heart rate). The head of the animals was fixed in a stereotactic frame, the skin of the head was incised in the sagittal line and the skin flaps extended. The parietal periosteum was removed with an electric coagulator and the parietal bone on both sides trepanned with a dental drill under liquid cooling until a thin layer of bone was left, which was carefully removed with a fine forceps without causing any lesion of the parietal dura mater. Then the animal was exsanguinated, the head was separated, skinned and freed from muscles as far as possible. The skull was cut horizontally in a plane below the temporal fossa separating the apical from the basal skull and the brain was carefully removed, so that the transparent dura mater of the trepanned parietal region was visible from inside the cranium. The cranium was washed in SIF for 30 min at room temperature. After checking with SIF that the dura mater spanning the parietal bone had no leak, the cranium was mounted in a glass well with the inner surface of the dura side up (Fig. 2A). The well was filled with 500 μL SIF, so that the outer side of the dura mater was diving into the solution. After 5 min the solutions were carefully removed without touching the tissue and were replaced by fresh SIF, which was again removed after 5 min. Then the well was refilled with SIF and 1 pM CGRP was filled into the cranium, both removed after 5 min, refilled and removed after further 15 min. The same procedure was performed using 1 nM CGRP. From each solution 100 μL were taken, 25 μL of EIA buffer were added and the samples deep-frozen until processing with the EIA as noted above. For physiological solutions the lower detection limit of the assay is 2 pg CGRP/mL according to the manufacturer’s information.

### Calculations and statistics

If at the same time point two samples of 25 μL CSF or plasma were collected and processed with the EIA, the two CGRP values were averaged prior to further calculations. Mean ± standard error of the mean (SEM) was calculated for corresponding data of repeated experiments. Statistical calculations were performed with Statistica 7.0 (StatSoft, Tusla, OK, USA). After testing normal distribution of values, ANOVA with repeated measurements and Fisher’s least significant difference (LSD) post hoc test were used to analyze the consecutive blood flow values at one-minute intervals and the CGRP concentration in SIF, blood and CSF samples. Differences were considered significant at *p* < 0.05. Flow values, which have no dimension, were normalized to the baseline for graphical representation.

## Results

### CGRP levels in the cerebrospinal fluid

**Fig. 3:**
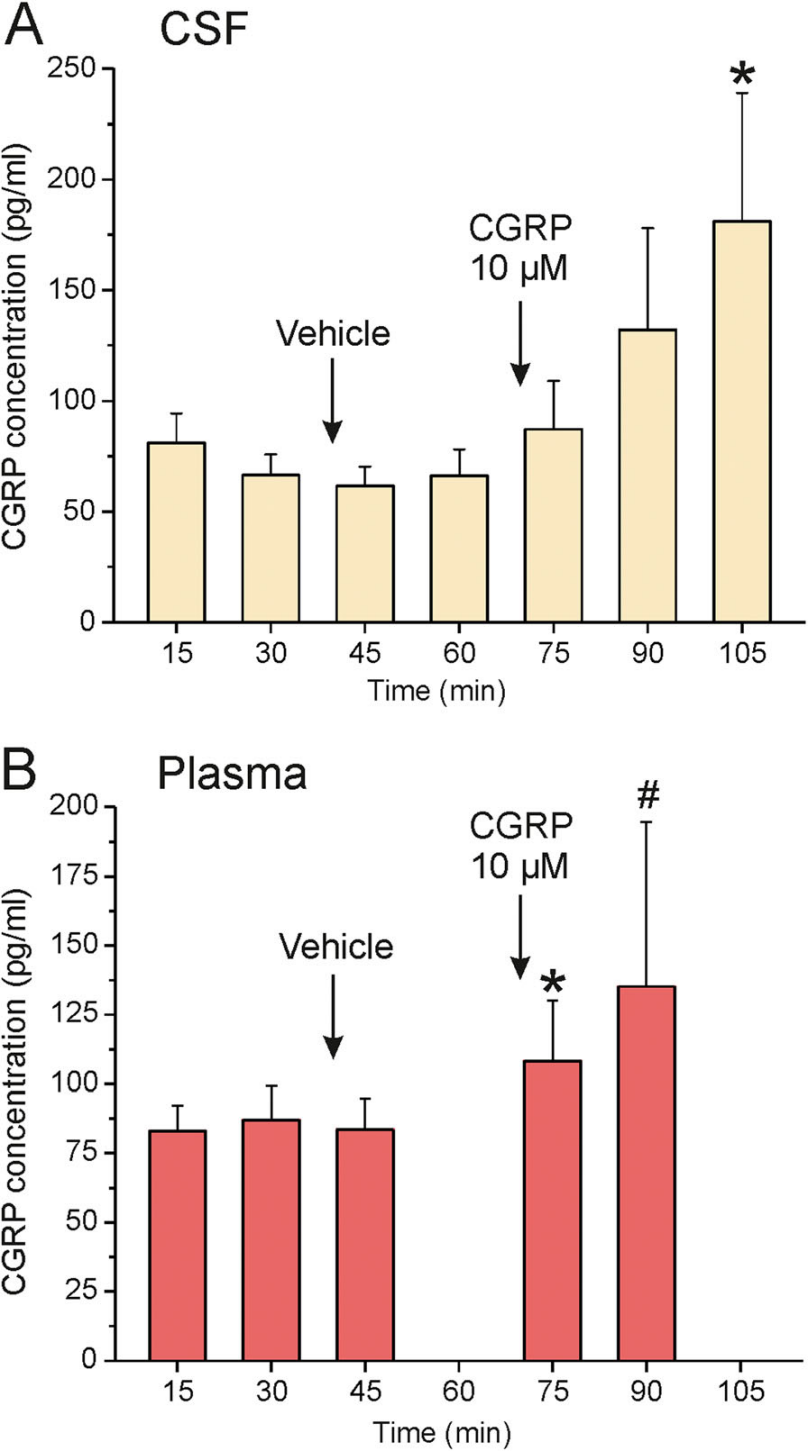
CGRP concentrations (means ± SEM) in samples of CSF (**A**, *n* = 10) and jugular plasma (**B**, *n* = 4-9) taken at the indicated time points after opening of the cisterna magna. * significant difference in A to values at 15-75 min, in B to values at 30 and 45 min, # to value at 75 min

CGRP in the CSF was determined in 10 experiments. CSF samples were obtained at intervals of 15 min (see Fig. 1C). The first sample taken 15 min after opening of the cisterna magna contained 76.7 ± 14.0 pg/mL CGRP, the second sample 64.0 ± 10.0 pg/mL CGRP (Fig. 3A). Five minutes after topical application of vehicle (SIF) onto the dura mater the CGRP level reached its lowest value with 57.9 ± 8.8 pg/mL before it continuously increased after CGRP at 10 μM was applied onto the dura. At 35 min after CGRP application the last CSF sample contained 191.5 ± 62.9 pg/mL CGRP. Repeated measures ANOVA indicated significant differences between the consecutive samples (*F* (6,54) = 2.52, *p* = 0.034). The LSD post-hoc test confirmed significant differences in CGRP concentration of the last sample compared to the first five samples (*p* = 0.004-0.022).

### CGRP levels in jugular plasma

CGRP in jugular blood plasma was determined in 10 experiments but only in seven experiments four or more blood samples with sufficient volume could be consecutively drawn from the jugular vein (Fig. 3B). The basal plasma CGRP level before application of vehicle (SIF) onto the dura mater was 87.0 ± 12.2 pg/mL, 5 min after application of vehicle (SIF) it was 83.5 ± 11.2 pg/mL. Five min after application of CGRP (10 μM) the CGRP plasma concentration was 108.3 ± 21.7 pg/mL, 15 min later 135.2 ± 59.5 pg/mL. Repeated measures ANOVA showed that the differences between the values just failed significance (*F* (4,4) = 4.59, *p* = 0.084) but the LSD post-hoc test indicated differences between the values before and after vehicle and the value 5 min after CGRP application (*p* < 0.02-0.04).

### Meningeal and cortical blood flow and blood pressure

**Fig. 4:**
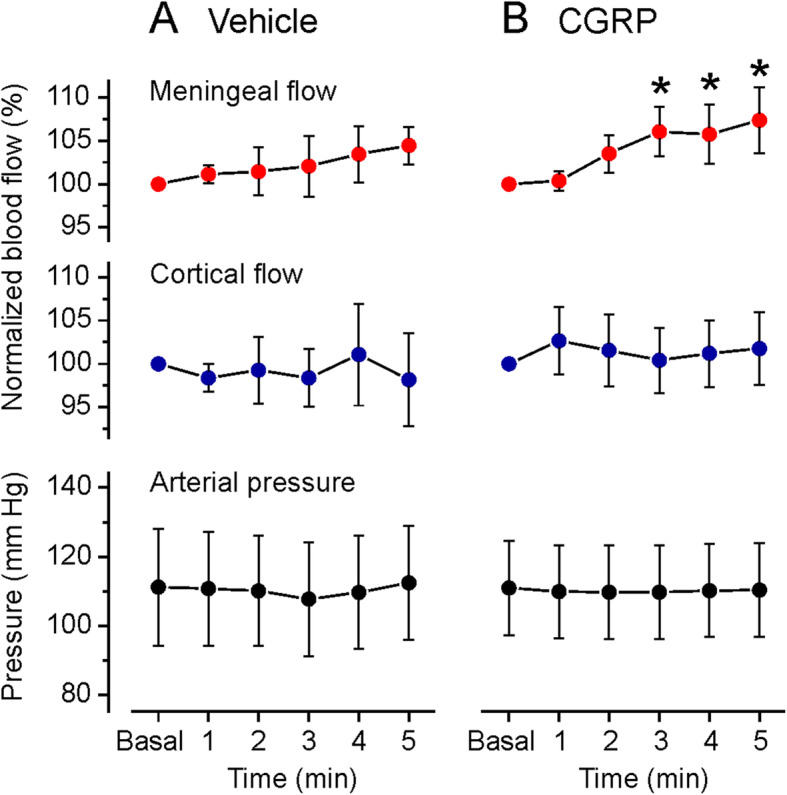
Meningeal and cortical blood flow normalized to baseline (Basal, the minute prior to application) of vehicle (SIF, A) and CGRP at 10 μM (B) onto the dura mater, and concomitant arterial pressure. Data are means ± SEM; * significant difference to baseline

To explore the vascular effects of topically applied CGRP, the meningeal blood flow around the middle meningeal artery and the cerebral blood flow around the middle cortical artery or the blood flow on the surface of the spinal medulla was recorded in 16 experiments, partly parallel to the blood sampling from the jugular vein. Meningeal blood flow was not recorded in experiments with CSF sampling, because an opening of the cisterna magna causes CSF leaking and a collapse of the subarachnoidal space below the dura mater which changes the geometry of the optical recording. Blood flow was continuously recorded and the minute intervals of 1 min before (baseline) to 5 min after the application of vehicle (SIF) and CGRP were analysed (see Fig. 1C). In 9 (meningeal flow recordings) and 7 experiments (cortical recording) 40 μL SIF (vehicle) was applied onto the dura mater; the meningeal and cortical blood flow remained without significant changes (repeated measures ANOVA, *F* (5,40) = 0.33, *p* = 0.89 for meningeal and *F* (5,25) = 0.56, *p* = 0.73 for cortical) (Fig. 4A). Application of CGRP at 1 μM in 5 experiments was not followed by a change in meningeal and cortical blood flow. After application of CGRP at 10 μM, the meningeal blood flow was continuously rising to 107.4 ± 3.8% (repeated measures ANOVA, *F* (5,45) = 2.66, *p* = 0.034) which was significant from the third minute post CGRP application compared to baseline (LSD post-hoc test, *p* = 0.013-0.029). In contrast, the cortical blood flow peaking at 102.6 ± 3.9% tended to increase but was not significantly different to baseline at any time (repeated measures ANOVA, *F* (5,35) = 1.14, *p* = 0.36) (Fig. 4B).

Topical application of SIF and CGRP (10 μM) did not lead to significant changes in systemic blood pressure measured in the femoral artery (repeated measures ANOVA, *F* (4,20) = 1.24, *p* = 0.33 for SIF and *F* (5,25) = 0.42, *p* = 0.83 for CGRP) (Fig. 4A, B).

### Ex vivo experiments

In 9 experiments, trepanned rat skulls were mounted in a glass well filled with SIF. CGRP concentrations were measured in the fluid within the skull, i.e. on the arachnoidal side of the dura mater, and in the buffered SIF outside in the glass well, i.e. on the periostal side of the dura (see Fig. 2). Prior to application, SIF without CGRP showed an apparent concentration of about 17 pg/mL, with addition of CGRP at 1 pM (3.8 pg/mL) the measured concentration was not significantly higher (about 18 pg/mL); adding CGRP at 1 nM (3.8 ng/mL) yielded a concentration of about 450 pg/mL. The discrepancy between expected and measured values is discussed below. Following application of SIF and the 1 pM CGRP concentration, arachnoidal CGRP concentrations were roughly half of the periostal CGRP concentrations, and CGRP levels tended to increase between 5 and 15 min of application, however, due to the high variation of data there was no statistically significant difference either between SIF/CGRP application nor between 5 and 15 min incubation (repeated measures ANOVA, *F* (7,56) = 1.71, *p* = 0.125). Following application of the 1 nM CGRP concentration, CGRP levels inside the skull after 5 min were more than double as high as the levels in the glass well (*F* (3,24) = 15.05, *p* < 0.001) with a decreasing difference after 15 min. In summary, the spontaneous release of CGRP seems to be higher on the periostal side (outside) compared to the arachnoidal side (inside) of the dura mater and picomolar concentrations of CGRP do not significantly contribute to the collected CGRP. Second, nanomolar CGRP concentrations can only partly be recovered in the samples and seem to diffuse only slowly through the dura mater into the glass well.

## Discussion

We have measured CGRP concentrations in the jugular blood and the CSF following application of a defined high concentration (10 μM) of this neuropeptide onto the exposed parietal dura mater. The purpose was to draw conclusions about the outflow of CGRP into these compartments. As a functional control of the presence of CGRP within the dura mater and in the CSF of the subarachnoidal space we measured meningeal and cerebral blood flow. We found an increase in CGRP concentration in jugular plasma and increasing meningeal blood flow already 5-20 min after the application. In comparison, the diffusion of CGRP into the CSF seemed to be somewhat delayed, as CGRP levels were significantly increased only 35 min after CGRP application. There was also only a tendency towards an increase in cortical blood flow, which is very likely caused by CGRP flushing pial arterial vessels [13]. We conclude that CGRP diffuses readily from the dural surface into the connective tissue around dural arteries, the compartment where CGRP is naturally released from perivascular afferents. Then the CGRP is rapidly absorbed by capillaries and postcapillary dural vessels, which lack a brain-blood barrier (BBB), and drained with the blood via the meningeal sinuses and the facial and maxillary veins into the (external) jugular vein [51, 52].

### CGRP drained into jugular plasma and CSF

Compared to the high CGRP concentration applied onto the dura mater, the increase in CGRP in the jugular plasma and the CSF was very small. This may be due, at least in part, to some important limitations in our experiments. First, the jugular catheter was placed in proximal direction to collect blood from the internal jugular vein. Different to humans and cats, however, this vein is very thin in rat [52] and thus may transport little amounts of blood from intracranial tissues to the cranial vena cava (see Fig. 1A). The main outflow of blood collected in the sinus system is via the anterior jugular and the facial vein, branches of the external jugular vein, which have necessarily been blocked by the catheter in our experiments. In addition, there may have been considerable decomposition of CGRP in blood between CGRP application and sampling. The half-live of CGRP in circulating blood is in the range of 10 min [53, 54], corresponding data from CSF have not been published. As in the present study, in previous experiments with a similar set-up CGRP levels in CSF were higher in the CSF compared to jugular blood after stimulation of the exposed dura mater with depolarizing concentrations of potassium chloride [13].

### Blood flow increases induced by CGRP

In accordance with earlier studies [55, 56], the high concentration of 10 μM CGRP applied to the dura mater caused only mild increases in meningeal blood flow and no significant increase in cortical flow. There is striking discrepancy compared with the high sensitivity of isolated (human) cerebral arteries to CGRP with a half-maximal relaxation effect of about 0.1-1 nM [57, 58] or vasodilatation by 80% of (rat) meningeal arteries caused by infusion of 1 μg/kg CGRP, which is in the same molar range when calculated for its distribution in the extracellular compartments [59, 60]. There is also discrepancy to the strong vasodilatory effect of electric field stimulation of the dura mater caused by local CGRP release [55, 61, 62]. Therefore, we assume that the local concentration around meningeal arteries of CGRP released from afferent fibres upon their stimulation is higher than the concentration that reaches the blood vessels after topical administration of CGRP, even at the relatively high concentration of 10 μM. That means, on the other hand, that only a small fraction of the CGRP applied onto the dura mater reaches the dural arteries, not to mention the pial arteries below the dura, where the measured CGRP concentration in the range of 100-200 pg/mL (around 20-40 pM) is clearly lower than the concentration causing half-maximal relaxation as mentioned above. The reason for this discrepancy is unclear. Possibly the mass of CGRP is degraded in the dura or taken up from postcapillary vessels before reaching the arterial smooth muscle cells to induce vasodilatation and increased blood flow.

### CGRP penetration through the dura mater

**Fig. 5:**
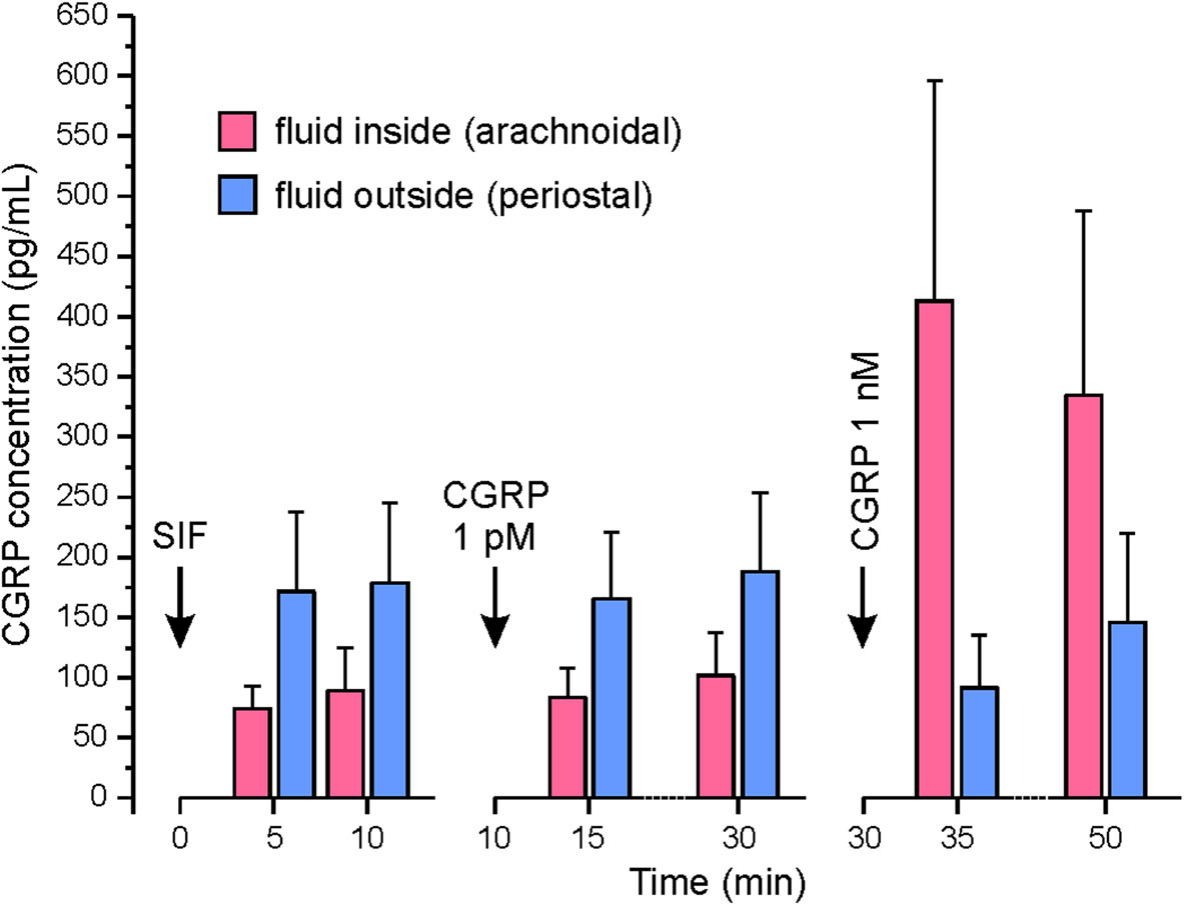
CGRP concentrations measured in the cranium (arachnoidal side) and on the outside (periostal side) of the dura mater (*n* = 9) after application of vehicle (SIF) and CGRP at concentrations of 1 pmol/L and 1 nmol/L.

Although in the CSF the increase in CGRP levels was somewhat delayed, the final concentrations were clearly higher than those found in the jugular plasma. The CSF was taken from the cisterna magna above the medullary brainstem, so that the delay may be a matter of the slow CSF flow from the subarachnoidal space underlying the exposed parietal dura to the medulla. However, the question is if and to which extent CGRP accumulating around trigeminal afferents in the dura mater diffuses into the CSF of the subarachnoidal space. The innermost layer of the dura mater, the “subdural neurothelium” or “neurothelial cell layer”, is a thin epithelial-like layer [33, 63]. The first layer below the dura, the outermost part of the arachnoid, is composed of epithelial-like “arachnoidal barrier cells” which are interconnected by tight junctions limiting diffusion of larger molecules [64]. When the brain with the adhering pia mater is removed from the skull, the leptomeninx is separated leaving the arachnoid barrier attached to the dura mater. To examine how permeable for CGRP the rat dura mater is, we developed an ex vivo preparation, in which we applied different concentrations of CGRP on the arachnoidal (inner) side and measured the CGRP concentration in the bath solution on the periostal (outer) side. After application of CGRP at 1 ng/mL only a small fraction of this concentration was measured in the bath solution outside, suggesting that the arachnoidal barrier cell layer is indeed a diffusion barrier for CGRP. A clear limitation of this approach is the fact that the measured concentrations in the solution filled into the cranium were also significantly lower than expected. Part of the CGRP may have been degraded in the dura mater indicated by a decrease in re-found CGRP concentrations with the time (see Fig. 5, right panel).

Another interesting phenomenon was visible after the application of CGRP at the very low concentration of 1 pg/mL. The CGRP concentration measured in the solutions on both sides of the dura was clearly higher than the applied concentrations, which may mainly be due to a spontaneous CGRP release from the dura but also the skull and some rests of tissue diving into the extracellular solution (see Fig. 5, left panels). Assuming that this is the main reason, a higher amount of CGRP is released from the periostal side of the dura (and from the bone) compared to the arachnoidal side, again indicating that there is a diffusion barrier covering the arachnoidal side of the dura mater.

## Conclusions

Taken together, we suggest that CGRP released from meningeal afferents in the cranial dura mater is partly eliminated in the dura, partly absorbed by postcapillary meningeal vessels and found in the venous outflow. Although the dura with the outer arachnoid layer is a diffusion barrier for CGRP, parts of the neuropeptide may also appear in the CSF in a delayed manner. Increased CGRP levels in the CSF have been sporadically reported for chronic but not episodic headaches/migraine [47]. Experimentally it was shown that after intracisternal application of noxious mediators the bulk of CGRP found in the CSF is released from primary meningeal afferents [10]. Different to our study, in this experimental study as well as in migraine, part of the CGRP in the CSF may origin from afferents innervating pial and cerebral blood vessels, which cannot absorb the released neuropeptides due to their blood brain barrier, so that it is accumulated in the CSF [31]. However, because of the high dilution and partial degradation of CGRP, its concentration in the CSF as well as in circulating blood plasma is far too low to cause an effective dilatation of pial or extracranial arteries nor could it contribute to sensitization of trigeminal nociceptors.

## Data Availability

All data generated and analysed during this study are included in this article.

## Abbreviations

ANOVA: Analysis of variance
BBB: Blood-brain barrier
cAMP: Cyclic adenosine monophosphate
CGRP: Calcitonin gene-related peptide
CSF: Cerebrospinal fluid
EIA: Enzyme immunoassay
SIF: Synthetic interstitial fluid
